# Presence of eating disorder symptoms in patients with obsessive-compulsive disorder

**DOI:** 10.1186/s12888-020-2457-0

**Published:** 2020-01-30

**Authors:** Lasse Bang, Unn Beate Kristensen, Line Wisting, Kristin Stedal, Marianne Garte, Åse Minde, Øyvind Rø

**Affiliations:** 1 Regional Department for Eating Disorders, Division of Mental Health and Addiction, Oslo University Hospital, P.O. Box 4956, Nydalen, N-0424 Oslo, Norway; 2 Specialized out-patient unit for OCD-spectrum Disorders, Division of Mental Health and Addiction, Oslo University Hospital, Oslo, Norway; 3 Specialized Outpatient Unit for Eating Disorders, Division of Mental Health and Addiction, Oslo University Hospital, Oslo, Norway; 4 Division of Mental Health and Addiction, Institute of Clinical Medicine, University of Oslo, Oslo, Norway

**Keywords:** Obsessive-compulsive disorder, Eating disorders, Anorexia nervosa, Bulimia nervosa, Comorbidity

## Abstract

**Background:**

Obsessive-compulsive disorder (OCD) is common in patients with eating disorders (EDs). There is a lack of research investigating the presence of ED symptoms among patients with OCD, despite concerns that many of these patients may be at high risk for EDs. Our objective was to assess the presence of ED symptoms in patients receiving treatment for OCD.

**Methods:**

Adult patients with OCD (*n* = 132, 71% females) and controls (*n* = 260, 90% females) completed the Eating Disorder Examination-Questionnaire (EDE-Q) at admission to a specialized OCD outpatient unit. A small subset of patients (*n* = 22) also completed the EDE-Q 3-months after end of treatment.

**Results:**

At the group-level, mean EDE-Q scores did not differ significantly between female patients and controls. However, female patients compared to controls were significantly more likely to score above the EDE-Q cut-off (23% vs. 11%) and have a probable ED (9% vs. 1%), indicating elevated rates of ED symptoms in the clinical range. There was no evidence of elevated rates of ED symptoms in male patients, though sample sizes were small. Preliminary follow-up data showed that certain ED symptoms improved significantly from admission to 3-month follow-up.

**Conclusions:**

Our findings suggest that while ED symptoms are not generally elevated in female patients with OCD, a considerable subset of female patients may have a clinical ED or be at high risk of developing one. Clinicians should be alert to ED symptoms in female patients with OCD, and our findings raise the issue of whether ED screening of female patients with OCD is warranted.

## Background

It has long been noted that patients with eating disorders (EDs) exhibit obsessive-compulsive traits [1], and obsessive-compulsive personality disorder co-occurs in 14–30% of patients [2]. Obsessive-compulsive disorder (OCD) is also frequently comorbid with EDs [3], and as many as 20–60% of patients with EDs have a lifetime history of OCD [4–6], although prevalence estimates vary widely [3, 7]. Studies [8, 9] have also shown that OCD predicts future development of anorexia nervosa (AN). The observed overlap between OCD-related conditions and EDs has led to the suggestion that these disorders are related and lie on the same spectrum [1, 10–12].

Less research has been devoted to map the prevalence of EDs among patients with OCD, despite concerns that this may be high [3]. Available studies have estimated that between 3 and 13% of patients has a lifetime history of an ED [13–17]. Lifetime ED rates are higher among female compared to male patients [14, 16, 18]. Less is known about how many patients with OCD have a *current* comorbid ED, but estimates range between 1 and 10% [13, 14, 18, 19]. Again, estimates are higher for females, and the few available studies report that between 7 and 18% of female and 0–5% of male patients have a current comorbid ED [14, 18]. As studies are limited by lack of control groups, the extent to which ED prevalence is higher in this patient group compared to non-patient populations is unclear. Also, few studies have adopted a dimensional approach to ED symptoms in OCD, which can be a valuable approach to broadly characterize ED risk in these patients. One study administered a self-report screening measure for EDs to patients with OCD, and reported that 18–34% of patients with OCD scored above the screening cut-offs for an ED [18]. These initial findings indicate that a significant proportion of patients may be at high risk of developing EDs, but more studies are needed.

Little is known about the course and outcomes of ED symptoms among patients with OCD. Preliminary evidence suggest that during treatment of patients with ED, OCD and ED symptoms improve in parallel [20]. However, it is unclear if ED symptoms similarly improve following treatment for OCD. In a longitudinal study, Micali and colleagues [19] reported that 1% of young patients with OCD had an ED at the time of admission to treatment, but at follow-up after treatment (on average 5 years later) 13% had an ED. Those with an ED were more likely to be female and to have persistent OCD at follow-up. These initial findings suggest clinicians should be alert to ED symptoms in patients with OCD, as many patients may be at high risk of developing an ED after treatment.

In summary, more studies describing the presence of ED symptoms among patients with OCD are needed. Existing studies are limited by lack of control groups and follow-up data. The current study therefore aimed to assess the presence of ED symptoms among patients with OCD. We hypothesized that ED symptoms would be more prevalent among patients. We also present preliminary 3-month follow-up data after end of treatment for a subset of female patients.

## Methods

### Participants

The sample (total *n* = 392) consisted of 132 patients with OCD (94 females and 38 males) and 260 controls (233 females and 27 males). Patients were recruited from the specialized OCD-team at Oslo University Hospital (Norway). In the study period this team offered both individual exposure and response prevention treatment over 8–12 weeks, and the Bergen 4-day treatment in group-format [21]. The Bergen 4-day treatment is a concentrated exposure and response prevention treatment delivered in groups of 3–6 patients by the same number of therapists over four consecutive days. Patients completed the self-report measures in paper-and-pencil format at admission, before treatment had started. All patients had a primary ICD-10 OCD diagnosis. Of note, some patients with a very low body weight (e.g. due to AN) are not offered treatment at this unit (this decision is based on clinical evaluation and not on any specific weight-threshold).

As a comparison group, we recruited controls from the general community. We first sent an invitation to participate along with the self-report measures in paper-and-pencil form to the home addresses of 400 individuals (between ages 18 and 40) randomly selected from the Norwegian Population Registry. However, only 45 (response-rate = 14%) returned the self-report measures, probably due to the fact that participants were required to post the self-report measures back to us. To acquire more controls we shared an invitation to participate on Facebook together with a link to an electronic (online) version of the self-report measures. The invitation to participate was similar to the one used during recruitment of participants from the Norwegian Population Registry. A total of 215 controls completed the electronic self-report measures. We compared controls recruited through the Norwegian Population Registry (who completed self-report measures in paper-and-pencil form) to controls recruited through Facebook (who completed self-report measures in electronic form). There were no significant differences in age or scores on any of the self-report measures (all *p*’s > .05). Previous studies have similarly shown that scores obtained from self-report measures do not differ between paper-and-pencil and electronic forms, in both patient [22] and non-patient populations [23]. The two control groups were therefore combined.

As the number of patients available to us during the study period was limited and the expected effect sizes uncertain, we performed sensitivity analyses to investigate the range of effect sizes we would have sufficient power to detect. We expected to be able to include 80–100 patients. To increase power, we aimed to recruit twice the amount of controls (160–200). Sensitivity analyses showed that with these sample sizes we would have 80% power to detect Cohen’s *d* of 0.34–0.39 and odds ratios of 2.34–2.54. We found this level of sensitivity acceptable, but aimed to include as many participants as possible. We anticipated the vast majority of participants would be female, and we were able to include the desired number of female participants (i.e. > 80 patients and > 160 controls). However, as a sizable number of males also participated (38 patients and 27 controls), we chose to include them in separate analyses. We recognize that the male sample sizes are small, and analyses of these likely underpowered. Given the scarcity of reports of ED symptoms among males, we nonetheless report these analyses in the hope that they will inform future studies.

A follow-up was not originally planned for this study, but we were able to perform one on a subset of patients to explore the longitudinal outcomes of ED symptoms. Patients who showed up to a clinical appointment with the OCD unit 3-months after end of treatment and had participated in the study were invited to the follow-up study. A subset of patients participated in the follow-up and were administered the same self-report measures they completed at admission. As only two males participated in the follow-up, we excluded these so the follow-up sample consisted entirely of females (*n* = 22). A larger sample was not possible to attain, as the project period had ended and the OCD unit could not continue recruiting patients for the follow-up due to resource constraints (e.g. involvement in other research studies). Data-collection for the follow-up study was therefore ended before all participants eligible for inclusion could be invited. Analyses based on this follow-up sample should therefore be considered preliminary and are presented to inform future longitudinal studies. All patients in the follow-up sample had been treated with the Bergen 4-day treatment. The study was approved by the regional ethics committee in Norway (reference: 2013/1209). All participants provided written informed consent.

### Self-report measures

Participants completed the following self-report measures, all translated and back-translated to Norwegian.

The Eating Disorder Examination-Questionnaire (EDE-Q [24];) is a widely used 28-item self-report measure assessing attitudinal features of EDs and core ED behaviors during the past 28 days. Except for items probing the frequency of ED-related behaviors, responses are rated on a 7-point scale, with possible scores ranging from 0 to 6. The EDE-Q is comprised of four subscales: dietary restriction, eating concern, weight concern, and shape concern. These subscales are averaged to calculate the EDE-Q global score. The Norwegian version of the EDE-Q has demonstrated satisfactory psychometric properties [25], and a global score of 2.5 has been established as a cut-off threshold to discriminate between patients with ED and controls [26]. Excellent internal consistency was found in the present study for both controls (*α* = .94) and patients (*α* = .96).

The Obsessive-Compulsive Inventory-Revised (OCI-R) is a 18-item self-report measure assessing OCD symptoms during the past month [27]. Items are rated on a 5-point scale with possible scores ranging from 0 to 4. All items are summed to calculate the total score with values ranging from 0 to 72. The Norwegian version of the OCI-R has demonstrated satisfactory psychometric properties [28], and score of 21 has been established as a cut-off threshold to discriminate between patients with OCD and controls [27]. Good internal consistency was found in the present study for both controls (*α* = .89) and patients (*α* = .83).

### Analyses

The EDE-Q global and subscale scores were used to assess ED symptoms. We calculated how many in each group scored above the EDE-Q global cut-off threshold (> 2.5), which indicate clinical levels of ED symptoms. Furthermore, we assessed how many in each group had a probable ED, based on responses to specific EDE-Q items that correspond to diagnostic criteria (see Additional file 1: Table S1 for details) for AN and bulimia nervosa (BN). For AN this included: a) EDE-Q global score above the cut-off threshold, b) body mass index (BMI) < 18.5, c) intense fear of weight-gain, and d) overvaluation of body shape/weight on self-evaluation. For BN, this included: a) EDE-Q global score above the cut-off threshold, b) frequent binge-eating behaviors, c) frequent compensatory behaviors (e.g. purging behaviors such as self-induced vomiting), and d) overvaluation of body shape/weight on self-evaluation. Previous studies have also used the EDE-Q for diagnostic classification, and demonstrated its ability to identify AN and BN diagnoses [29–31]. Probable presence of binge-eating disorder was not assessed as the EDE-Q lacks questions corresponding to the diagnostic criteria for this disorder.

**Fig. 1:**
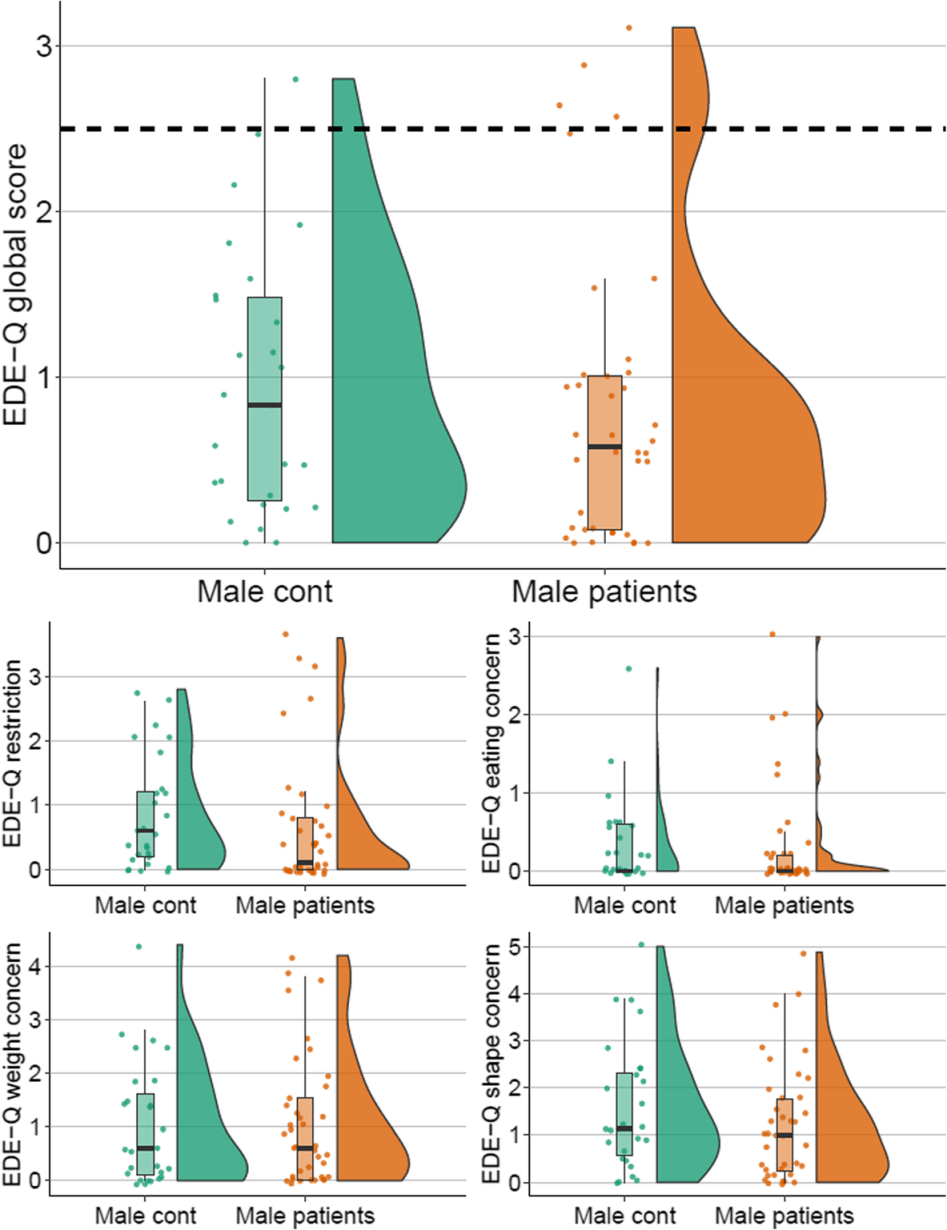
Raincloud plots showing the distributions of EDE-Q global and subscale scores in male groups. *Note*. Horizontal dashed line signifies the EDE-Q global cut-off threshold. Cont: Controls; EDE-Q: Eating Disorder Examination-Questionnaire

**Fig. 2:**
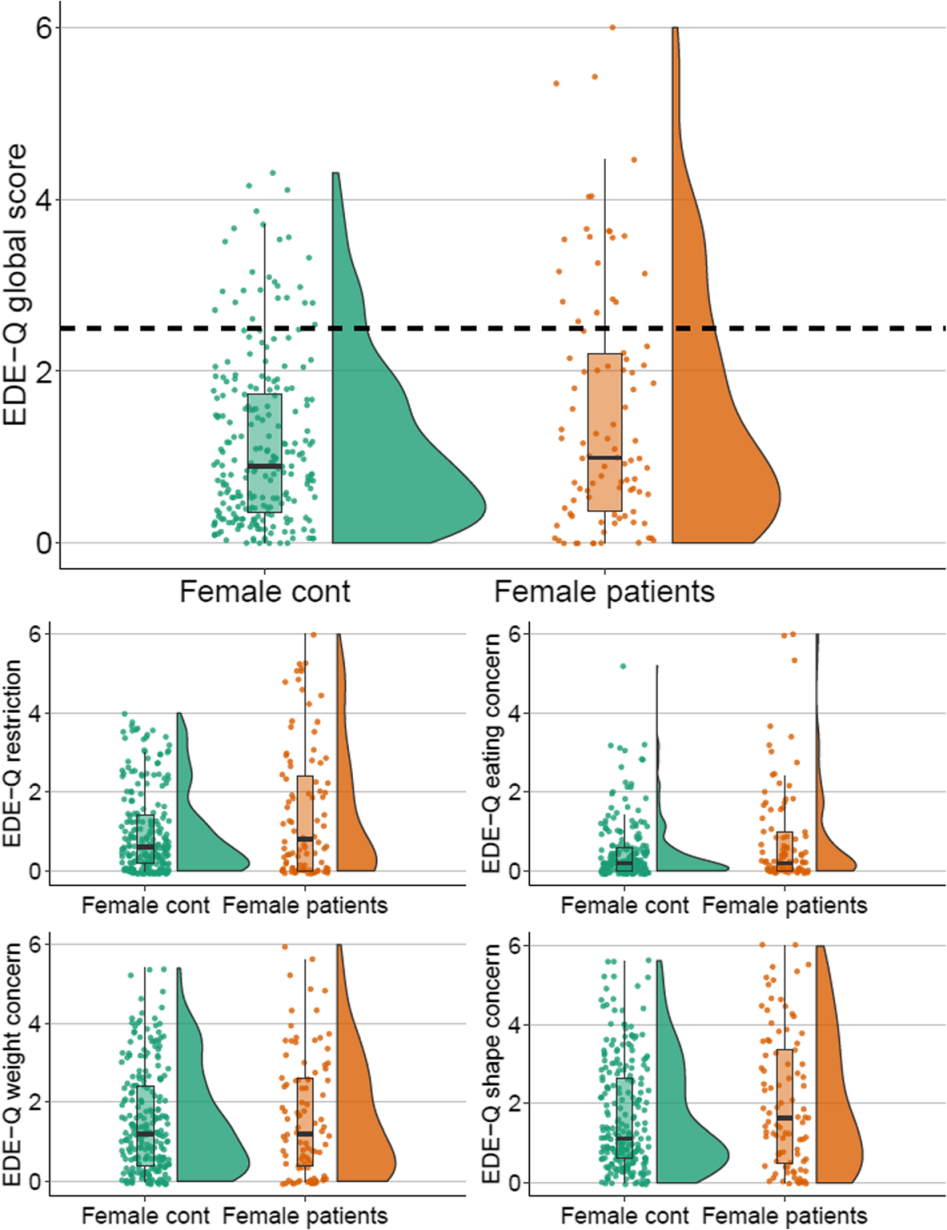
Raincloud plots showing the distributions of EDE-Q global and subscale scores in female groups. *Note*. Horizontal dashed line signifies the EDE-Q global cut-off threshold. Cont: Controls; EDE-Q: Eating Disorder Examination-Questionnaire

Between-group differences in age, BMI, and self-report measures at admission were investigated using t-tests. These variables were characterized by non-normal distributions according to visual inspections and normality-tests (see Figs. 1 and 2 and Additional file 1: Table S2). Also, a considerable number of outliers were identified (see Additional file 1: Table S3) using the median absolute deviation method [32]. While these outliers are of interest as they reflect individuals scoring in the extreme and clinical range of ED symptoms, they violate the assumptions underlying t-tests. Because of these issues, we used robust independent-samples Yuen t-tests for trimmed means [33], as implemented in the WRS2 R package [34]. This is a robust parametric version of the t-test, and provides better Type-I error control in situations of non-normality and heterogeneity of variances. We report robust Cohen’s *d* for these tests, following the method suggested by Algina and colleagues [35], and as implemented in the WRS2 package. Between-group differences on categorical variables at admission (EDE-Q cut-off threshold, probable ED, binging and purging presence) were tested using Wald *χ*^*2*^ tests (or Fisher’s exact test, where appropriate), for males and females separately. As females tend to report higher scores on ED-related measures [36, 37], male and female groups were analyzed separately. *P*-values for between-group analyses were adjusted according to a Bonferroni correction, to achieve a family-wise alpha-level of .05. These adjustments were performed for males and females separately. To explore associations between ED and OCD symptoms at admission, we calculated Spearman *r*_*s*_ correlations between the EDE-Q global and OCI-R total scores, for each group separately. For these correlations, *p*-values were Bonferroni-adjusted to achieve a family-wise alpha-level of .05.

For the follow-up sample, we performed dependent-samples Yuen t-tests to investigate changes in OCI-R and EDE-Q scores from admission to 3-month follow-up (using the WRS2 R package). For these comparisons we calculated Cohen’s *d*_*av*_ using the formula described by Cumming [38], and applied a Hedge’s *g* correction as described by Lakens [39]. Due to the low number of patients in the follow-up sample, we were not able to perform any additional analyses (e.g. *χ*^*2*^ tests). Considering the exploratory nature of these comparisons and the small sample size, results should be considered preliminary. No adjustment to alpha-levels were performed, and tests where *p* < .05 were deemed statistically significant.

All statistical analyses were two-tailed, and performed using R Studio version 1.2.1335 [40]. Raw data, as well as an R script to reproduce the main analyses described in this study are available at https://osf.io/qb8sd/ (doi: 10.17605/OSF.IO/QB8SD).

## Results

### Participant characteristics

For patients, self-reported mean age of onset of their disorder was 15.4 (*SD* = 6.6) years, and mean self-reported duration of illness was 13.9 (*SD* = 10.4) years. Mean number of self-reported previous treatments was 1.9 (*SD* = 2.3). The majority (69%) of patients scored above the OCI-R total cut-off threshold at admission. See Tables 1 and 2 and Figs. 1 and 2 for between-group comparisons for females and males, respectively. Female patients were significantly younger and characterized by significantly lower BMI compared to female controls. Male patients and male controls did not differ significantly in age or BMI. Unsurprisingly, female and male patients scored significantly higher on the OCI-R total compared to female and male controls.

**Table 1:** Comparisons between male patients and controls

Variable	OCD (*n* = 38)	Controls (*n* = 27)		Independent-samples Yuen t-test		
	*M (SD)*	*M (SD)*	Trimmed mean difference (95% CI)	*t*	*p*	Robust *d* (95% CI)
Age	33.92 (8.95)	32.44 (5.75)	0.70 (− 3.53; 4.94)	0.33	1.000	0.08 (− 0.38; 0.58)
BMI (kg/m^2^)	25.50 (4.47)	26.50 (4.75)	−0.86 (−2.95; 1.23)	− 0.79	1.000	− 0.23 (− 0.75; 0.31)
OCI-R total^b^	27.36 (12.91)	6.44 (8.09)	21.24 (14.61; 27.88)	6.93	<.002*	1.68 (1.22; 2.40)
EDE-Q global	0.82 (0.88)	0.94 (0.80)	−0.23 (− 0.65; 0.19)	−1.05	1.000	− 0.30 (− 0.95; 0.18)
EDE-Q restriction	0.64 (1.01)	0.84 (0.86)	−0.36 (− 0.87; 0.16)	− 1.43	0.770	− 0.42 (− 0.95; 0.14)
EDE-Q eating concern	0.32 (0.69)	0.34 (0.58)	− 0.12 (− 0.34; 0.10)	− 1.22	1.000	−0.36 (− 1.01; 0.14)
EDE-Q weight concern	1.07 (1.22)	1.02 (1.14)	−0.01 (− 0.62; 0.60)	−0.02	1.000	−0.01 (− 0.55; 0.48)
EDE-Q shape concern	1.24 (1.23)	1.58 (1.35)	−0.36 (− 1.01; 0.30)	− 1.07	1.000	− 0.28 (− 0.88; 0.23)
	Frequencies		*Χ*^*2*^ test			
	*n* (%)	*n* (%)	*p*			
Above EDE-Q cut-off	4 (10.5%)	1 (3.7%)	1.000^a^			
Have a probable ED	0 (0%)	0 (0%)	NA			
Have binged	5 (13.2%)	7 (25.9%)	0.638^a^			
Have purged	2 (5.3%)	1 (3.7%)	1.000^a^			

*Note*. All p-values have been adjusted according to a Bonferroni correction for multiple comparisons. BMI: Body mass index (kg/m^2^); CI: Confidence interval; *d*: Cohen’s robust *d* effect size; ED: eating disorder; EDE-Q: Eating Disorder Examination-Questionnaire; *M*: Means; NA: analysis not performed; OCD: Patients with obsessive-compulsive disorder; OCI-R: Obsessive Compulsive Inventory-Revised; *SD*: Standard deviation; *Χ*^*2*^: Wald *Χ*^*2*^ test. * Statistically significant. ^a^ Fisher’s exact test. ^b^ Data not available for one patient

**Table 2:** Comparisons between female patients and controls

Variable	OCD (*n* = 94)	Controls (*n* = 233)		Independent-samples Yuen t-test		
	*M (SD)*	*M (SD)*	Trimmed mean difference (95% CI)	*t*	*p*	Robust *d* (95% CI)
Age^a^	28.93 (8.62)	32.96 (7.00)	−5.64 (−7.30; −3.98)	−6.56	<.002*	−0.85 (−1.15; − 0.54)
BMI^b^ (kg/m^2^)	22.45 (3.37)	25.10 (4.85)	−2.21 (−3.12; − 1.30)	− 4.69	<.002*	− 0.55 (− 0.80; − 0.33)
OCI-R total^c^	28.60 (12.77)	5.88 (7.03)	23.99 (20.79; 27.20)	15.43	<.002*	3.12 (2.49; 3.82)
EDE-Q global^d^	1.51 (1.41)	1.16 (0.98)	0.26 (−0.10; 0.61)	1.42	0.825	0.22 (−0.06; 0.52)
EDE-Q restriction^d^	1.47 (1.68)	0.96 (1.05)	0.37 (−0.04; 0.78)	1.77	0.385	0.29 (−0.00; 0.62)
EDE-Q eating concern^d^	0.83 (1.27)	0.45 (0.74)	0.21 (−0.05; 0.47)	1.89	0.495	0.35 (0.06; 0.65)
EDE-Q weight concern^d^	1.68 (1.53)	1.53 (1.33)	0.14 (−0.28; 0.57)	0.64	1.000	0.09 (−0.17; 0.37)
EDE-Q shape concern^d^	2.07 (1.72)	1.68 (1.40)	0.40 (−0.10; 0.89)	1.57	0.550	0.23 (−0.05; 0.53)
	Frequencies		*Χ*^*2*^ test			
	*n* (%)	*n* (%)	*p*			
Above EDE-Q cut-off	21 (22.6%)	26 (11.2%)	.032*			
Have a probable ED	8 (8.7%)	3 (1.3%)	.010^e*^			
Have binged	21 (22.8%)	38 (16.3%)	.679			
Have purged	8 (8.8%)	14 (6.0%)	1.000			

*Note*. All p-values have been adjusted according to a Bonferroni correction for multiple comparisons. BMI: Body mass index (kg/m^2^); CI: Confidence interval; *d*: Cohen’s robust *d* effect size; ED: Eating disorder; EDE-Q: Eating Disorder Examination-Questionnaire; *M*: Means; NA: Analysis not performed; OCD: Patients with obsessive-compulsive disorder; OCI-R: Obsessive Compulsive Inventory-Revised; *SD*: Standard deviation; *Χ*^*2*^: Wald *Χ*^*2*^ test. * Statistically significant. ^a^ Data not available for one control. ^b^ Data not available for two patients. ^c^ Data not available for 12 patients. ^d^ Data not available for one patient. ^e^ Fisher’s exact test

### ED symptoms among patients with OCD at admission

Between-group comparisons showed no evidence of elevated ED symptoms among male patients compared to controls (see Table 1 and Fig. 1). Mean EDE-Q global and subscale scores did not differ significantly between male patients and controls. Four (11%) male patients scored above the EDE-Q cut-off, which was not significantly different from controls (4%). There were also no significant differences in rates of binging or purging behaviors. None of the male patients or controls had a probable ED.

Mean EDE-Q global and subscale scores did not differ significantly between female patients and controls (See Table 2 and Fig. 2), even when age and BMI were added as covariates. Rates of binging and purging behaviors were also not significantly different between groups. However, it was clear that a considerable proportion of female patients scored within the extreme and clinical spectrum of ED symptoms (e.g. see Fig. 2). Twice as many female patients scored above the EDE-Q cut-off compared to female controls (23% vs. 11%), and this difference was significant (see Table 2). Moreover, a significantly higher proportion of female patients compared to female controls were classified as having a probable ED (9% vs. 1%, see Table 2), although we underscore that these rates are based on a low number of positive cases (8 vs. 3) and should not be regarded as precise estimates. Among the female patients six were found to have probable BN, and two to have probable AN. These results indicate that while groups do not differ significantly in mean EDE-Q scores, female patients are more likely to screen positive for an ED and report a combination of ED symptoms indicative of a clinical ED.

Medium and significant positive correlations between the EDE-Q global and OCI-R total scores were evident for both female patients (*r*_s_ = .30, *p* = .030) and female controls (*r*_*s*_ = .32, *p* < .0005). These correlations were smaller and non-significant for male patients (*r*_*s*_ = .07, *p* = 1.0) and male controls (*r*_*s*_ = .23, *p* = 1.0). See Additional file 1: Figure S1 for scatterplots.

### ED symptoms among patients with OCD at 3-month follow-up

For the follow-up sample of patients, mean age was 29.0 (*SD* = 7.4) years. Approximately 29% of patients reported having received other psychological treatments since completing their OCD treatment. At follow-up compared to admission, patients reported large and significant reductions on the OCI-R total scores (see Table 3). Significant small reductions in the EDE-Q weight and shape concerns subscales were also evident (see Table 3). Scores on the EDE-Q global, restriction subscale, and eating concern subscale did not differ significantly between the two time-points. Given the small sample size, these results should be regarded as preliminary. Several patients reported clinical levels of ED symptoms at 3-month follow-up: three (14%) patients scored above the EDE-Q cut-off at 3-month follow-up, and these were also classified as having a probable ED (two with BN and one with AN). In total, nine (10%) of all female patients with OCD had a probable ED at some point during the study.

**Table 3:** Changes in EDE-Q and OCI-R scores from admission to 3-month follow-up

Variable	*n* = 22 Admission	*n* = 22 3-month follow-up		Dependent-samples Yuen t-test		
	*M (SD)*	*M (SD)*	Trimmed mean difference (CI)	*t (df)*	*p*	*g* (95% CI)
OCI-R total^a^	32.56 (14.09)	9.60 (7.39)	−22.79 (−31.33; −14.24)	−5.76 (13)	< .0005*	−1.92 (−3.03; −1.02)
EDE-Q global	1.80 (1.69)	1.38 (1.47)	−0.44 (−0.89; −0.00)	−2.11 (17)	.050	−0.25 (− 0.51; − 0.01)
EDE-Q restriction	1.51 (1.71)	1.20 (1.47)	−0.24 (− 0.84; 0.35)	−0.87 (17)	.395	−0.19 (− 0.52; 0.14)
EDE-Q eating concern	1.05 (1.79)	0.67 (1.49)	−0.37 (− 0.85; 0.12)	− 1.59 (17)	.131	− 0.22 (− 0.47; − 0.02)
EDE-Q weight concern	2.18 (1.72)	1.69 (1.62)	−0.60 (− 1.10; − 0.10)	−2.52 (17)	.022*	−0.28 (− 0.56; − 0.02)
EDE-Q shape concern	2.44 (1.94)	1.94 (1.84)	− 0.59 (− 1.09; − 0.09)	−2.49 (17)	.023*	−0.25 (− 0.50; − 0.03)

*Note*. All *p*-values are uncorrected for multiple comparisons. CI: Confidence interval; *df* = degrees of freedom; EDE-Q: Eating Disorder Examination-Questionnaire; *g*: Hedge’s *g* effect size; *M*: Means; OCI-R: Obsessive Compulsive Inventory-Revised; *SD*: Standard deviation. * Statistically significant. ^a^ Data not available for four patients at admission and two patients at 3-month follow-up

## Discussion

We showed that mean EDE-Q scores did not differ significantly between female patients and controls, but female patients were more likely to report ED symptoms in the clinical range. Our results suggest that while ED symptoms are not generally elevated in female patients with OCD, a considerable subset may have a clinical ED or be at high risk of developing one. In contrast, there was no evidence of elevated rates of ED symptoms in male patients.

In contrast to our hypothesis, mean EDE-Q scores did not differ significantly between female patients and controls. This suggests that as a group, female patients with OCD are not characterized by particularly high levels of ED symptoms, as one might have suspected based on the high comorbidity rates and the purported link between OCD and EDs. Studies have shown that OCD symptoms are elevated in patients with EDs [41, 42], even following recovery [43, 44]. It is possible that OCD symptoms are more prevalent among patients with EDs than the converse, but more studies are needed to address this. Of note, patients relative to controls reported slightly higher EDE-Q global and subscale scores, and we cannot rule out that statistically and clinically significant differences between groups exist and would be evident with larger sample sizes.

While ED symptoms were not elevated at the group-level, our results showed that female patients were significantly more likely than controls to report ED symptoms in the clinical range. Approximately 23% of female patients scored above the EDE-Q screening cut-off, and 9% were found probable to have an ED; rates which were significantly higher than controls. These findings show that a considerable proportion of female patients report ED symptoms in the clinical range. These patients may have a clinical ED or be at high risk of developing one, and may be in need of ED-specific treatment at some point. A previous study found that 18–34% (depending on cut-off score) of female patients with OCD screened positive for an ED [18], and another that 7% of female patients had a co-occurring ED [14]. Although these studies used ED measures other than the EDE-Q, their rates are comparable to the ones we report. Importantly, as our study included a comparison group, we were able to show that these rates are significantly higher than controls.

In contrast to female patients, there was no evidence of elevated levels of ED symptoms among male patients, and none had a probable ED. Our findings are in line with previous research demonstrating an association between female gender and the presence of ED symptoms in OCD [3, 14, 18, 19]. However, few males participated in our study, and analyses were likely underpowered. Larger studies of male patients with OCD are clearly needed.

Our findings show that a considerable proportion of female patients with OCD report ED symptoms in the clinical range, and may either have an ED or be at high risk of developing one. These findings suggest clinicians should be alert to ED symptoms in female patients with OCD. There is evidence to suggest that comorbid EDs are unrecognized in the majority of treatment-seeking patients with anxiety-related (including OCD) disorders [45]. ED symptoms are often ego-syntonic and associated with shame, and may not be readily disclosed by patients. It may therefore be advisable to assess ED-related features in young female patients with OCD, including weight fluctuations, eating behaviors, purging behaviors, and preoccupation with weight and shape. Our findings also showed that ED and OCD symptoms are positively correlated in female patients and controls, similar to what other studies have reported [10, 46]. This suggests that presence of ED symptoms may be associated with more severe OCD features. Our findings, along with those of previous studies, raise the issue of whether ED screening of female patients with OCD is warranted. ED screening may identify individuals who have an ED or are at high risk of developing one. ED screening measures are available and can be used for this purpose, for example the EDE-Q or the SCOFF [47]. It is worth noting that we were unable to assess presence of binge-eating disorder, which is a common disorder among both females and males [48]. We might therefore have under-estimated the prevalence of probable EDs among patients. Also, as some patients with a very low body-weight (e.g. due to AN) are not offered treatment at the OCD unit where we recruited patients, the prevalence of AN-related symptoms among patients with OCD could be higher than that reported in our study.

At 3-month follow-up, certain ED symptoms improved significantly, while others did not. These preliminary findings suggest some ED symptoms (concern about ones weight and shape) are ameliorated following OCD treatment, perhaps due to the general mental health benefits of treatment. Several female patients still reported clinical levels of ED symptoms at follow-up; 13% of the follow-up sample scored above the EDE-Q cut-off and had a probable ED. However, we stress that our follow-up sample was small, and larger studies are needed to accurately characterize the outcomes of ED symptoms among patients with OCD.

A number of limitations of our study are worth noting. First, our study relied on self-report measures of ED symptoms, which are not optimal for diagnostic classification. Our results therefore cannot address rates of *clinical* EDs. However, the use of self-reports enabled us to investigate the proportion of participants scoring above a validated screening cut-off, to identify at-risk individuals. Also, while patients completed the self-report measures in paper-and-pencil form, the majority of controls completed them in electronic form. This might have contributed to differences in EDE-Q scores between groups, though our analyses indicated that control participants’ EDE-Q and OCI-R scores did not differ between self-report forms. Another limitation pertains to the gender distribution of the control group, which was overwhelmingly female. The representativeness of the few control males who participated is therefore unclear. As few males participated, the male-specific analyses were likely underpowered. Future studies including more males are clearly needed to more precisely characterize this group. Last, the follow-up sample was small, and results from these analyses should be regarded as preliminary.

## Conclusions

In conclusion, our findings suggest that while ED symptoms are not generally elevated in female patients with OCD, a considerable subset of female patients may have a clinical ED or be at high risk of developing one. Clinicians should therefore be alert to ED symptoms in female patients with OCD, and our findings raise the issue of whether ED screening of female patients with OCD is warranted. Future studies with larger sample sizes are needed to further investigate the presence and outcomes of ED symptoms among patients with OCD.

## Supplementary information

**Additional file 1: Table S1.** EDE-Q criteria for a probable eating disorder. **Table S2.** Results from normality tests. **Table S3.** Outliers detected with median absolute deviation method. **Figure S1.** Scatterplots showing the association between OCI-R total and EDE-Q global scores.

## Data Availability

The datasets supporting the conclusions of this article are available in the OSF repository; doi: 10.17605/OSF.IO/QB8SD, https://osf.io/qb8sd/.

## Abbreviations

AN: Anorexia nervosa
BMI: Body mass index
BN: Bulimia nervosa
ED: Eating disorder
EDE-Q: Eating disorder examination-questionnaire
OCD: Obsessive-compulsive disorder
OCI-R: Obsessive-compulsive inventory-revised
