# Supplementary material for: Presence of eating disorder symptoms in patients with obsessive-compulsive disorder
Source: BMC Psychiatry. 2020 Jan 30;20:36. doi: 10.1186/s12888-020-2457-0 (PMC6993325; doi:10.1186/s12888-020-2457-0)
Supplement: Supplementary file 1 — Additional file 1: Table S1. EDE-Q criteria for a probable eating disorder. Table S2. Results from normality tests. Table S3. Outliers detected with median absolute deviation method. Figure S1. Scatterplots showing the association between OCI-R total and EDE-Q global scores. [file 12888_2020_2457_MOESM1_ESM.docx]

**Table S1. EDE-Q criteria for a probable eating disorder**

| Probable ED | Criteria |
| --- | --- |
| AN | a) EDE-Q global score > 2.5.  b) BMI < 18.5.  c) EDE-Q item 10 >= 4 (fear of weight-gain).  d) At least one of EDE-Q items 22 or 23 >= 4 (overvaluation of weight or shape). |
| BN | a) EDE-Q global score > 2.5.  b) EDE-Q item 15 >= 4 (days with binge-eating).  c) At least one of EDE-Q items 16, 17, or 18 >= 4 (episodes of compensatory behaviors).  d) At least one of EDE-Q items 22 or 23 >= 4 (overvaluation of weight or shape).  e) Do not satisfy criteria for AN. |

*Note*. AN: Anorexia nervosa; BMI: Body mass index (kg/m^2^); BN: Bulimia nervosa; ED: Eating disorder; EDE-Q: Eating Disorder Examination-Questionnaire.

**Table S2. Results from normality tests.**

| Variable | Shapiro-Wilkes | Anderson-Darling | Jarque-Berra |
| --- | --- | --- | --- |
| Female controls |  |  |  |
| Age | < .001 | < .05 | < .001 |
| BMI | < .001 | < .001 | < .001 |
| OCI-R total | < .001 | < .001 | < .001 |
| EDE-Q global | < .001 | < .001 | < .001 |
| EDE-Q restriction subscale | < .001 | < .001 | < .001 |
| EDE-Q eating concern subscale | < .001 | < .001 | < .001 |
| EDE-Q weight concern subscale | < .001 | < .001 | < .001 |
| EDE-Q shape concern subscale | < .001 | < .001 | < .001 |
| Male controls |  |  |  |
| Age | .360 | .487 | .552 |
| BMI | .139 | .077 | .381 |
| OCI-R total | < .001 | < .001 | < .001 |
| EDE-Q global | < .05 | < .05 | .292 |
| EDE-Q restriction subscale | < .05 | < .05 | .145 |
| EDE-Q eating concern subscale | < .001 | < .001 | < .001 |
| EDE-Q weight concern subscale | < .001 | < .001 | < .05 |
| EDE-Q shape concern subscale | < .05 | < .05 | .164 |
| Female patients |  |  |  |
| Age | < .001 | < .001 | < .001 |
| BMI | < .05 | < .05 | < .05 |
| OCI-R total | .444 | .723 | .559 |
| EDE-Q global | < .001 | < .001 | < .001 |
| EDE-Q restriction subscale | < .001 | < .001 | < .05 |
| EDE-Q eating concern subscale | < .001 | < .001 | < .001 |
| EDE-Q weight concern subscale | < .001 | < .001 | < .05 |
| EDE-Q shape concern subscale | < .05 | < .001 | < .05 |
| Male patients |  |  |  |
| Age | .486 | .561 | .558 |
| BMI | < .05 | < .05 | < .001 |
| OCI-R total | < .05 | < .05 | .296 |
| EDE-Q global | < .001 | < .001 | < .05 |
| EDE-Q restriction subscale | < .001 | < .001 | < .001 |
| EDE-Q eating concern subscale | < .001 | < .001 | < .001 |
| EDE-Q weight concern subscale | < .001 | < .001 | < .05 |
| EDE-Q shape concern subscale | < .05 | < .05 | < .05 |

*Note*. Results from three normality tests. P-values < .05 and < .001 indicate data is non-normally distributed. BMI: Body mass index (kg/m^2^); EDE-Q: Eating Disorder Examination-Questionnaire; OCI-R: Obsessive Compulsive Inventory-Revised.

**Table S3. Outliers detected with median absolute deviation method.**

| Variable | Median | MAD | Limits of acceptable values | Number of extremely low outliers | Number of extremely high outliers |
| --- | --- | --- | --- | --- | --- |
| Female controls |  |  |  |  |  |
| Age | 34.0 | 5.9 | 16.2; 51.8 | 0 | 5 |
| BMI | 24.1 | 4.2 | 11.6; 36.5 | 0 | 8 |
| OCI-R total | 4.0 | 4.4 | -9.3; 17.3 | 0 | 16 |
| EDE-Q global subscale | 0.9 | 0.9 | -1.8; 3.6 | 0 | 6 |
| EDE-Q restriction subscale | 0.6 | 0.9 | -2.1; 3.3 | 0 | 13 |
| EDE-Q eating subscale | 0.2 | 0.3 | -0.7; 1.1 | 0 | 32 |
| EDE-Q weight subscale | 1.2 | 1.2 | -2.4; 4.8 | 0 | 4 |
| EDE-Q shape subscale | 1.1 | 1.3 | -2.7; 5.0 | 0 | 7 |
| Male controls |  |  |  |  |  |
| Age | 33.0 | 5.9 | 15.2; 50.8 | 0 | 0 |
| BMI | 26.2 | 4.5 | 12.6; 39.8 | 0 | 0 |
| OCI-R total | 4.0 | 4.4 | -9.3; 17.3 | 0 | 2 |
| EDE-Q global | 0.8 | 0.9 | -1.9; 3.6 | 0 | 0 |
| EDE-Q restriction subscale | 0.6 | 0.9 | -2.1; 3.3 | 0 | 0 |
| EDE-Q eating subscale | 0.0 | 0.00 | 0.0; 0.0 | 0 | 13 |
| EDE-Q weight subscale | 0.6 | 0.9 | -2.1; 3.3 | 0 | 1 |
| EDE-Q shape subscale | 1.1 | 1.3 | -2.7; 5.0 | 0 | 1 |
| Female patients |  |  |  |  |  |
| Age | 27.0 | 5.9 | 9.2; 44.8 | 0 | 7 |
| BMI | 22.0 | 3.3 | 12.1; 31.9 | 0 | 2 |
| OCI-R total | 28.0 | 11.9 | -7.6; 63.6 | 0 | 0 |
| EDE-Q global | 1.0 | 1.2 | -2.6; 4.6 | 0 | 3 |
| EDE-Q restriction subscale | 0.8 | 1.2 | -2.8; 4.4 | 0 | 10 |
| EDE-Q eating subscale | 0.2 | 0.3 | -0.7; 1.1 | 0 | 22 |
| EDE-Q weight subscale | 1.2 | 1.8 | -4.1; 6.5 | 0 | 0 |
| EDE-Q shape subscale | 1.6 | 1.9 | -3.9; 7.2 | 0 | 0 |
| Male patients |  |  |  |  |  |
| Age | 32.5 | 9.6 | 3.6; 61.4 | 0 | 0 |
| BMI | 24.6 | 3.8 | 13.3; 35.9 | 0 | 1 |
| OCI-R total | 25.0 | 15.6 | -21.7; 71.7 | 0 | 0 |
| EDE-Q global | 0.6 | 0.7 | -1.6; 2.8 | 0 | 2 |
| EDE-Q restriction subscale | 0.1 | 0.2 | -0.3; 0.5 | 0 | 14 |
| EDE-Q eating subscale | 0.0 | 0.0 | 0.0; 0.0 | 0 | 13 |
| EDE-Q weight subscale | 0.6 | 0.9 | -2.1; 3.3 | 0 | 4 |
| EDE-Q shape subscale | 1.0 | 1.1 | -2.3; 4.3 | 0 | 1 |

*Note*. BMI: Body mass index (kg/m^2^); EDE-Q: Eating Disorder Examination-Questionnaire; OCI-R: Obsessive Compulsive Inventory-Revised.

**Figure S1. Scatterplots showing the association between OCI-R total and EDE-Q global scores.**


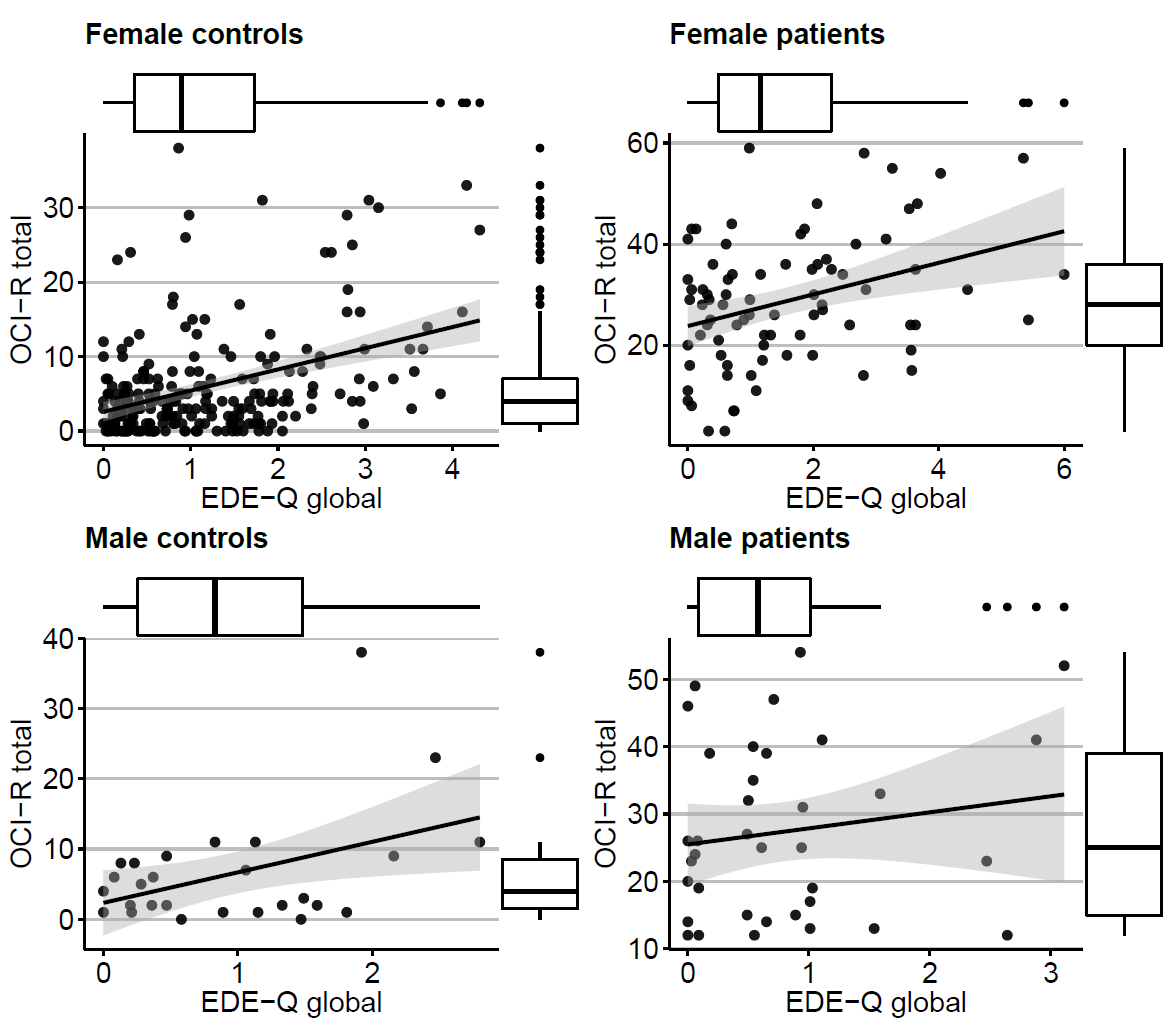


*Note*. EDE-Q: Eating Disorder Examination-Questionnaire; OCI-R: Obsessive Compulsive Inventory-Revised.
